# Supplementary figures and images for: Actively replicating gut bacteria identified by 5-ethynyl-2’-deoxyuridine (EdU) click chemistry and cell sorting
Source: Gut Microbes. 2023 Feb 23;15(1):2180317. doi: 10.1080/19490976.2023.2180317 (PMC9980609; doi:10.1080/19490976.2023.2180317)

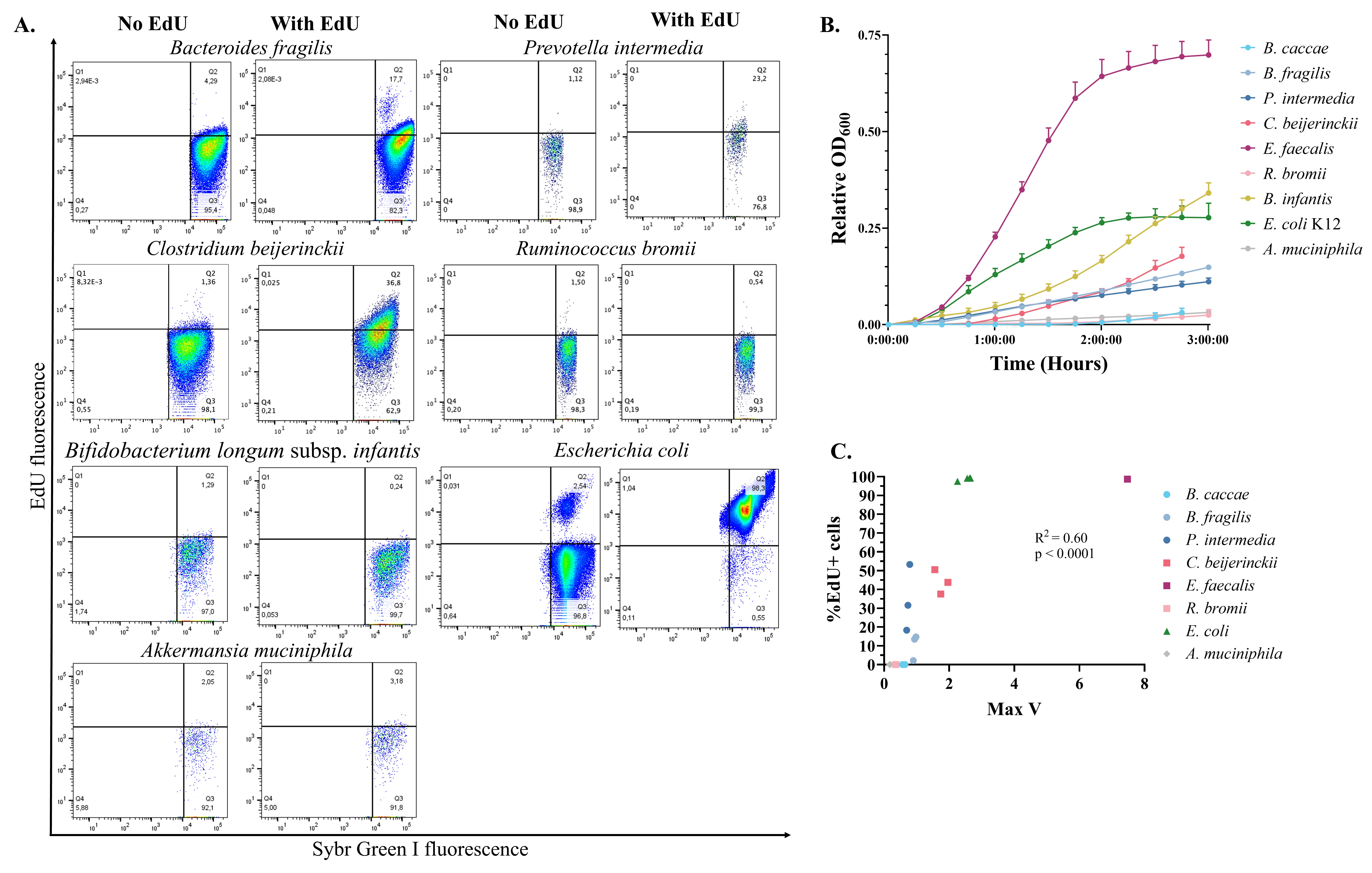

Supplement: Supplemental Material [file KGMI_A_2180317_SM8183.zip › FigS1.jpg]

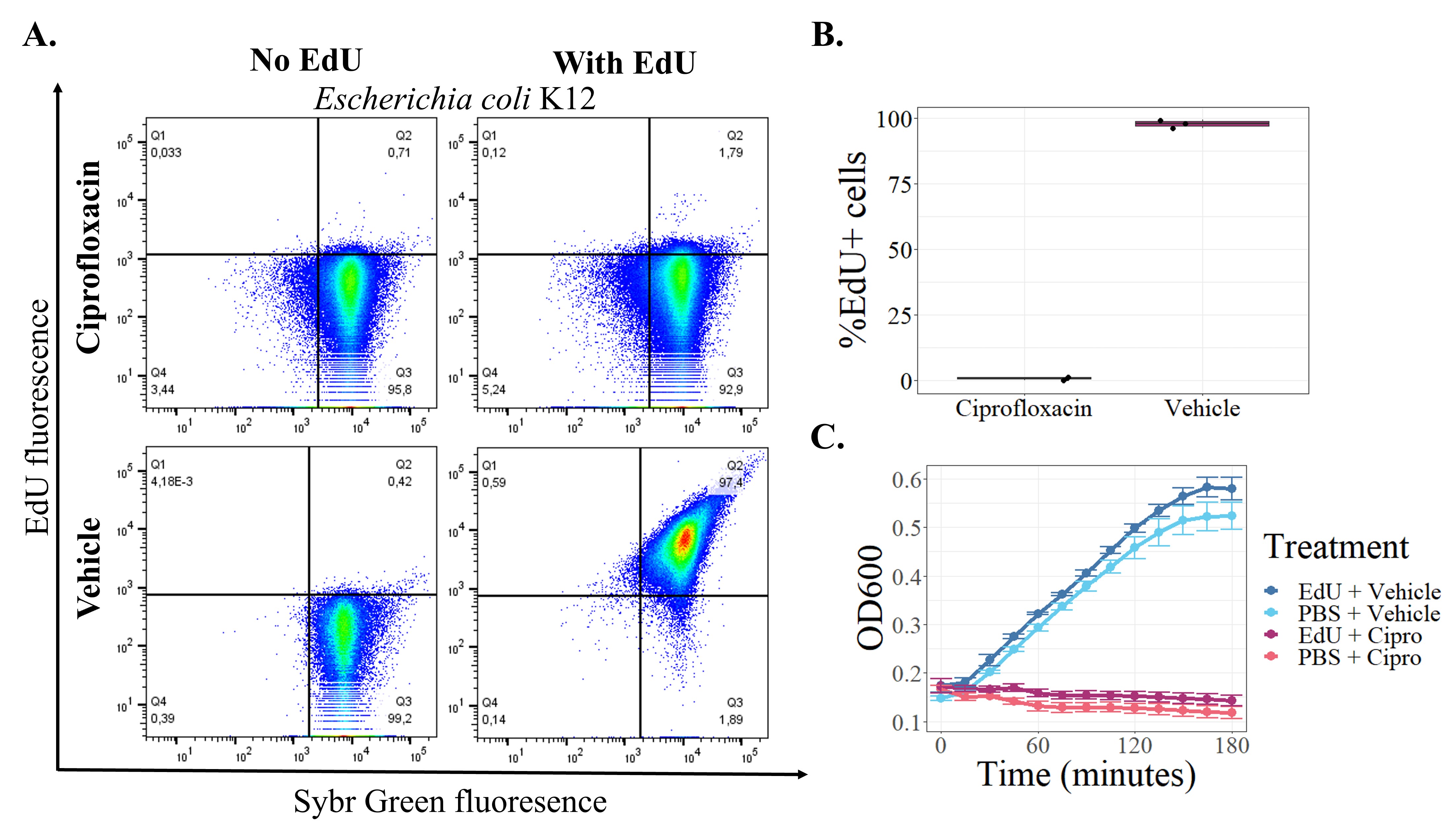

Supplement: Supplemental Material [file KGMI_A_2180317_SM8183.zip › FigS3.jpg]

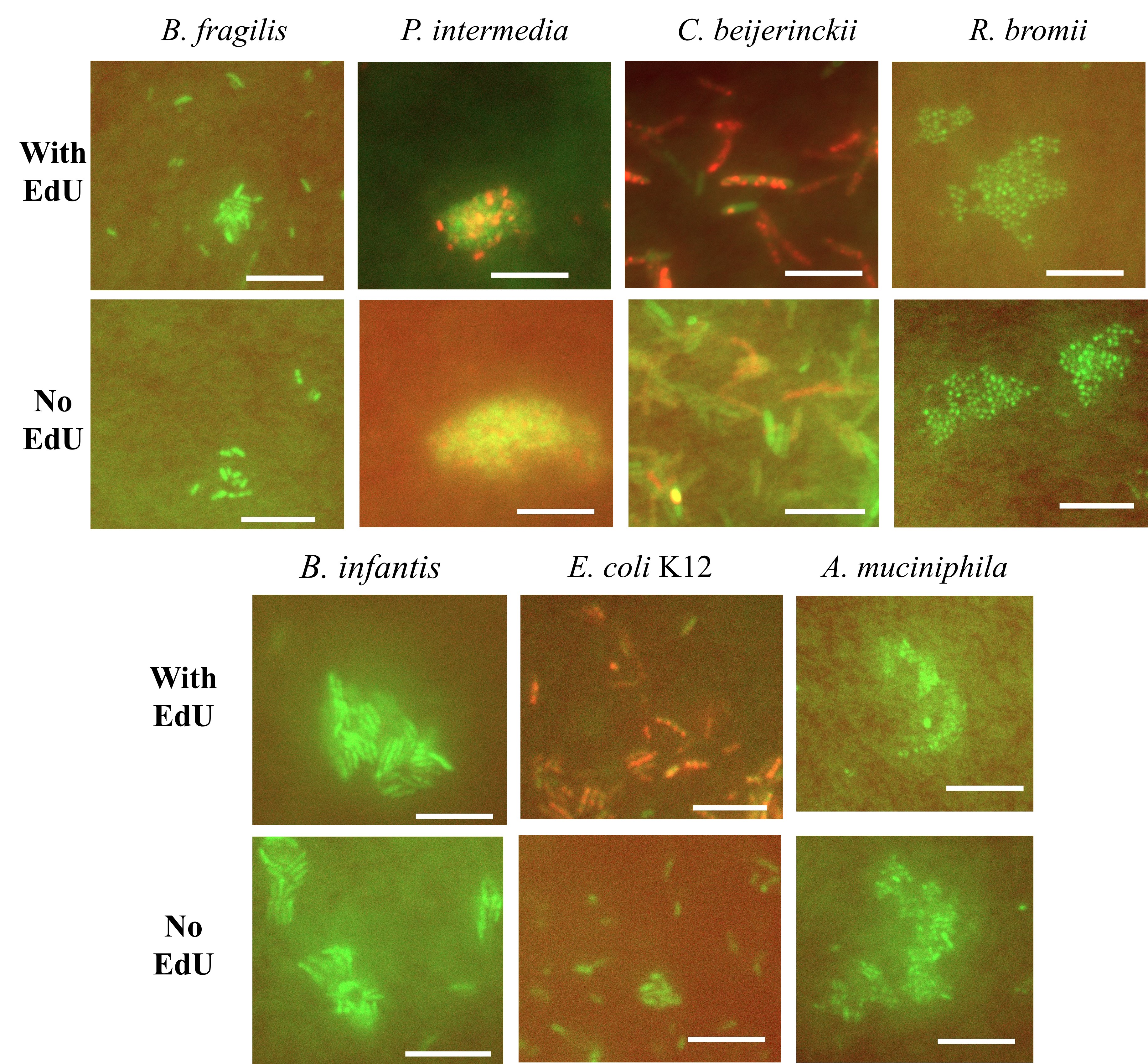

Supplement: Supplemental Material [file KGMI_A_2180317_SM8183.zip › FigS4.jpg]

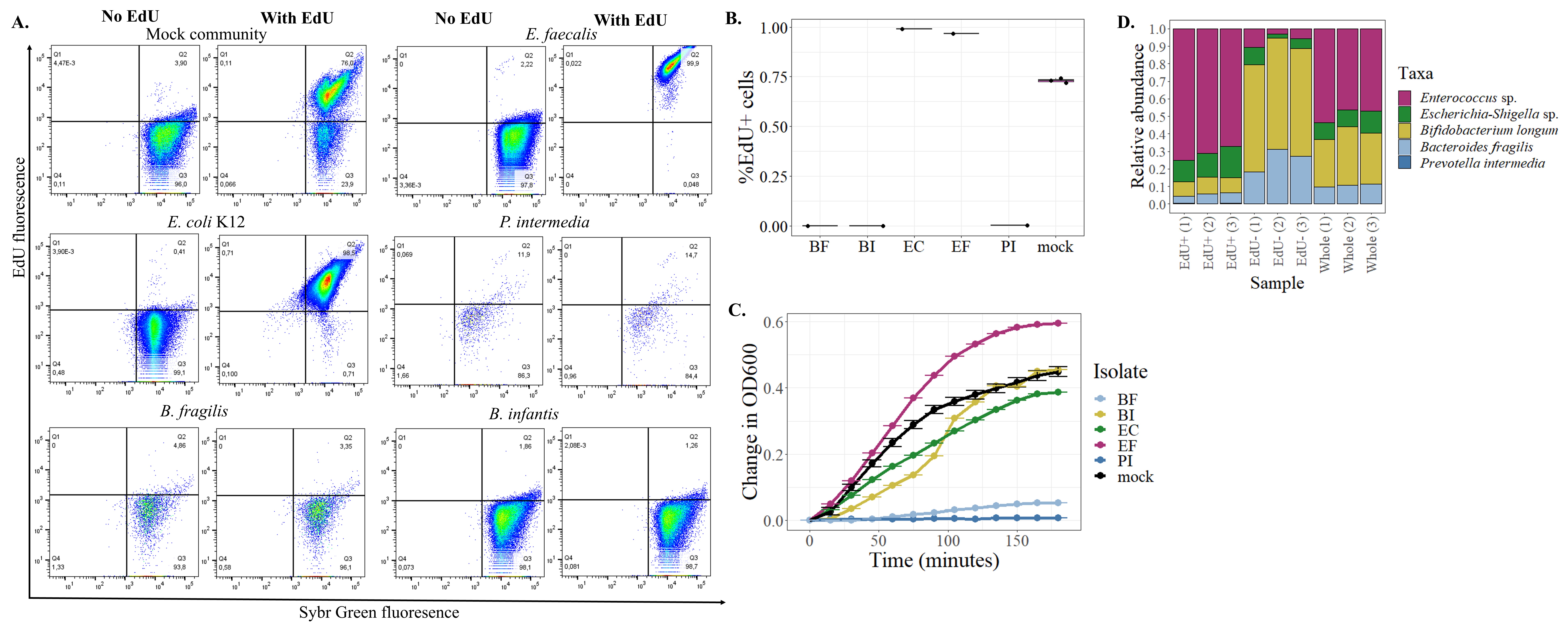

Supplement: Supplemental Material [file KGMI_A_2180317_SM8183.zip › FigS5.jpg]

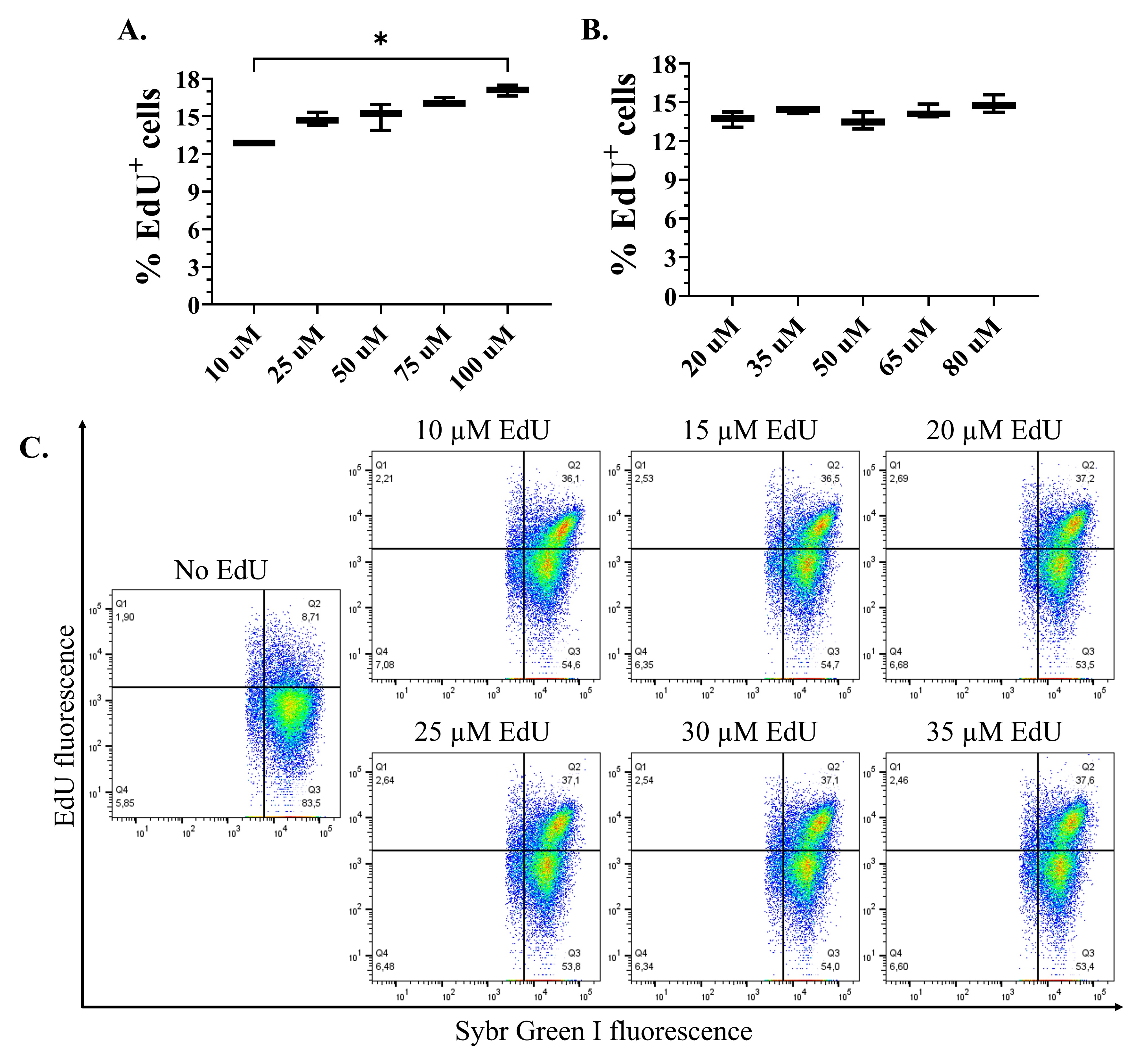

Supplement: Supplemental Material [file KGMI_A_2180317_SM8183.zip › FigS6.jpg]

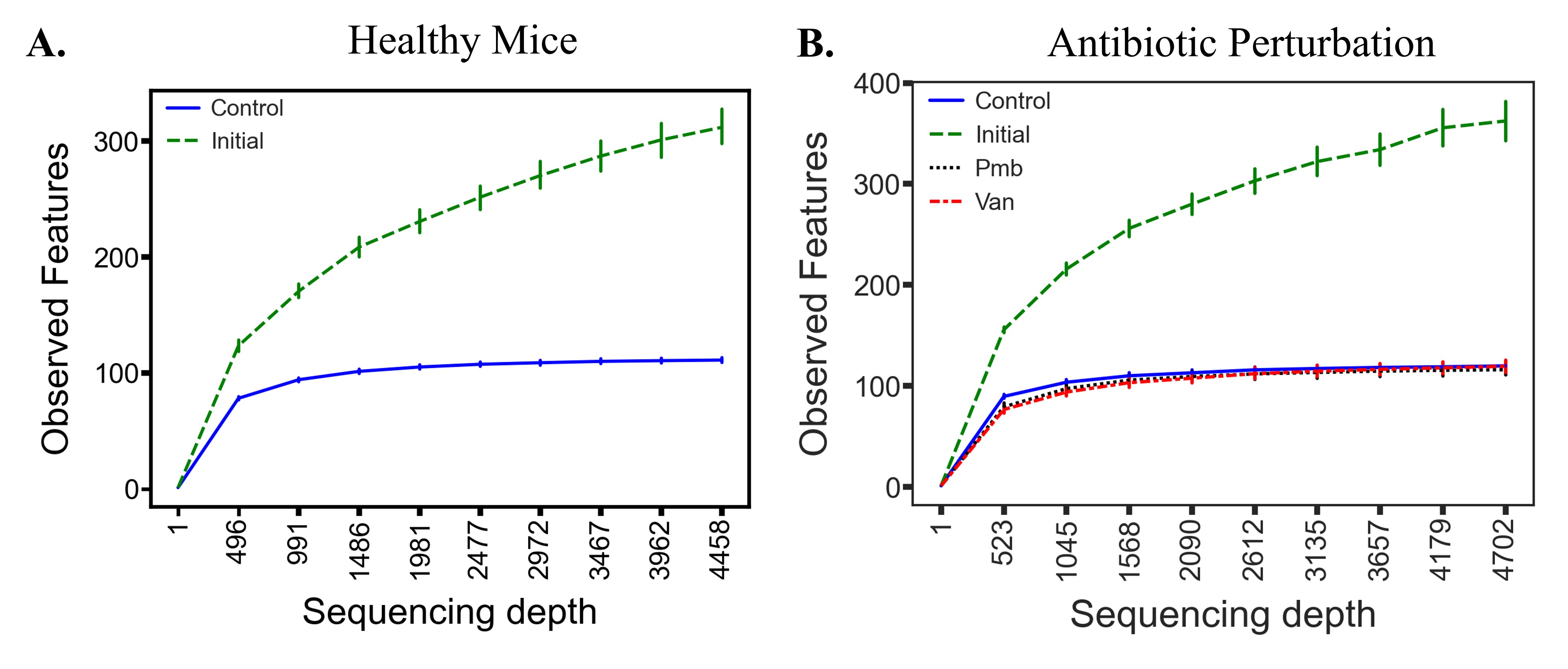

Supplement: Supplemental Material [file KGMI_A_2180317_SM8183.zip › FigS7.jpg]

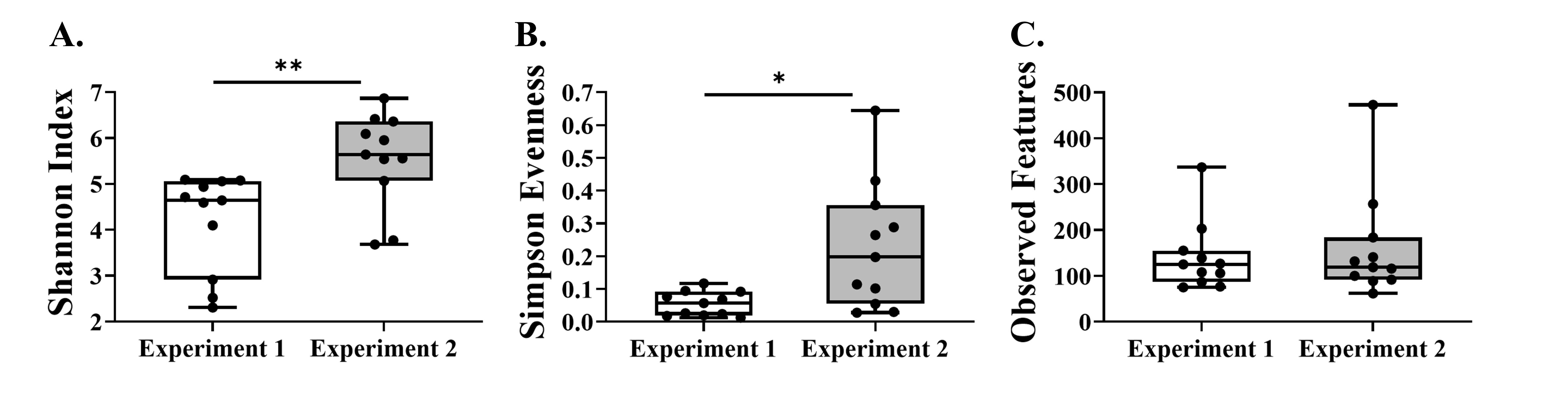

Supplement: Supplemental Material [file KGMI_A_2180317_SM8183.zip › FigS8.jpg]
